# A Multi-Parameter Integrated Sensor Based on Selectively Filled D-Shaped Photonic Crystal Fiber

**DOI:** 10.3390/ma15082811

**Published:** 2022-04-12

**Authors:** Dan Yang, Tiesheng Wu, Yiping Wang, Weiping Cao, Huixian Zhang, Zhihui Liu, Zuning Yang

**Affiliations:** 1 Guangxi Key Laboratory of Wireless Broadband Communication and Signal Processing, School of Information and Communication, Guilin University of Electronic Technology, Guilin 541004, China; dyang_guet@163.com (D.Y.); weipingc@guet.edu.cn (W.C.); hxzhang_guet@163.com (H.Z.); zhihuil_guet@163.com (Z.L.); zuning_yang@163.com (Z.Y.); 2 Key Laboratory of Optoelectronic Devices and Systems of Ministry of Education and Guangdong Province, College of Optoelectronic Engineering, Shenzhen University, Shenzhen 518060, China; ypwang@szu.edu.cn; 3 Guangdong and Hong Kong Joint Research Centre for Optical Fiber Sensors, College of Optoelectronic Engineering, Shenzhen University, Shenzhen 518060, China

**Keywords:** multi-parameter, D-shaped photonic crystal fiber, sensor, selective filling

## Abstract

We propose and numerically investigate a multi-parameter integrated sensor based on a selectively filled D-shaped photonic crystal fiber (PCF). The simple structure can be used to comprehensively detect refractive index, magnetic field, temperature, and voltage. According to the surface plasmon resonance and directional coupling effect, the PCF is coated with a gold nano-film to detect the refractive index of the external environment. In addition, magnetic fluid (water-based Fe_3_O_4_), toluene, and nematic liquid crystal (NLC E7) are selectively filled into different cladding air holes of the D-shaped PCF to realize the different sensing of the magnetic field, temperature, and voltage. The measurement of refractive index, magnetic field, temperature, and voltage are independent of each other, so these four parameters can be measured simultaneously. The sensing characteristics of the proposed structure are investigated systematically by the finite element method. The results show that the sensitivities of refractive index, magnetic field, temperature, and voltage are 4600 nm/RIU, 1.375 nm/Oe, 15.143 nm/°C, and 0.971 nm/V, respectively. The presented design based on materials selectively filled with D-shaped PCF might enable promising application in multi-parameter optical sensing.

## 1. Introduction

Surface plasmon resonance (SPR) is a physical optical phenomenon occurring on the interface between metal and dielectric [1,2,3,4]. It is extremely sensitive to changes in permittivity of the metal and dielectric, and the structural shape [5,6,7,8]. This feature provided a highly sensitive optical sensing technology that can be used for real-time detection and has been widely used in biochemical sensing applications [9]. In recent years, the PCF sensor based on the SPR effect has attracted extensive attention [10,11,12,13,14,15,16,17,18,19,20]. In 1993, Jorgenson et al. first proposed the SPR fiber optic sensor with an RI resolution of 7.5 × 10^−4^ RIU [21]. In 2019, Bing et al. proposed and analyzed a D-shaped PCF sensor with elliptical air holes with a high RI sensitivity of 10,200 nm/RIU [22]. Amiri et al. studied the graphene oxide effect on the performance of the D-shaped SPR fiber sensor. The sensitivity of this structure is up to 833.33 nm/RIU, and the response of graphene oxide shows an increase in sensitivity [23]. In 2021, Tian et al. discussed a quasi-D-shaped PCF biosensor whose RI sensitivity is up to 12,000 nm/RIU when the RI range is 1.21~1.32, and the maximum resolution is 8.33 × 10^−6^ RIU [24]. At the same time, Bing et al. designed a highly sensitive dual-sample channel PCF sensor, which can be used for synchronous detection. The sensor adopted a structure with a large D-shaped air hole and a long vertical distance from the fiber core. The corresponding wavelength sensitivity was 16,200 and 15,800 nm/RIU [25]. Mitu et al. designed a two-dimensional PCF RI sensor based on SPR for pregnancy detection. This sensor can obtain a good sensitivity response in urine observation, and the sensitivity response, linewidth sensitivity, and resolution are 68,051.344 nm/RIU, 314 RIU^−1^, and 9.39 × 10^−6^ RIU, respectively [26]. Ayyanar et al. proposed a phase-change material, reconfigurable biosensor based on PCFs for tunable and enhanced RI sensing in the near-infrared region. The average bulk RI sensitivities of the crystalline and amorphous phases obtained by this sensor are 17,600 nm/RIU and 8000 nm/RIU, respectively [27].

However, the above SPR fiber sensors can only detect one parameter. Suppose multiple parameters can be detected in one sensor simultaneously, and the sensing of various parameters is independent of each other; in that case, the integrated performance of the sensor can be optimized effectively for real-environment applications. In 2018, Dong et al. designed a dual-parameter sensor based on a D-shaped fiber for detecting magnetic field and temperature simultaneously [28]. In 2019, Ying et al. designed a liquid crystal (LC)-filled D-shaped PCF sensor for RI and temperature detection. When the RI range is 1.0~1.6 and the temperature range is 15~50 °C, the RI sensitivity and the maximum temperature sensitivity of the sensor are 2275 nm/RIU and 9.09 nm/°C, respectively [29]. In 2020, Jiang et al. proposed a polarization filter based on an LC-filled PCF, which has the characteristics of miniaturization and high bandwidth, and the crosstalk can reach 177.34 and 203.43 dB when the wavelengths are 1.31 and 1.55 µm, respectively. Moreover, the bandwidth is as high as 1200 nm in the wavelength range of 1.2~2.4 µm [30]. In 2021, Taghizadeh et al. theoretically and experimentally investigated a magnetic field sensor based on a tapered PCF Mach–Zehnder interferometer assisted by magnetic fluid (MF). The sensor’s magnetic field and strain sensitivity are −0.072 nm/mT and 1 pm/με, respectively [31]. In the same year, Mo et al. designed a selectively filled D-shaped PCF sensor to simultaneously measure magnetic field intensity and temperature. Its sensitivity can be up to 0.14274 nm/Oe and −0.229 nm/°C, respectively [32]. It is well known that SPR sensors are widely used in various biological systems from proteins, oligonucleotides, oligosaccharides, and lipids to small molecules, phages, viral particles, and cells. However, through SPR detection it is difficult to distinguish non-specific adsorption, sensitive to interference factors such as temperature, electromagnetic field, or sample composition. Therefore, it is especially important to design a class of SPR fiber optic sensors that can simultaneously detect ambient temperature, electric field and voltage.

In this work, we propose a multi-parameter integrated sensor based on a selectively filled D-shaped PCF. It is designed according to the principle of the directional coupling effect caused by the selective filling of the PCF and the SPR effect caused by polishing and plating gold nano-film on the surface of the PCF. The sensor characteristics are numerically investigated using COMSOL Multiphysics software. We analyzed the effects of RI coefficient, magnetic field intensity, temperature, voltage, air hole diameter, air hole diameter filled with different materials, hole spacing, gold nano-film thickness, and polishing depth on the features of the D-shaped PCF in detail and found that these parameters have significant effects on the strength of the mode coupling. The air hole diameter in the structural parameters has a more significant effect on temperature sensitivity. The results show that the average refractive index sensitivity of the sensor is up to 4600 nm/RIU with the refractive index range of 1.33~1.42. The magnetic field sensitivity can be up to 1.375 nm/Oe for the magnetic field range of 75~275 Oe. The temperature sensitivity reaches 15.143 nm/°C at the range of 15~50 °C. When the voltage range is 0~70 V, the voltage sensitivity is 0.971 nm/V. The sensor proposed in this paper can well break through the limitation of single-parameter measurement of traditional sensors and achieve the purpose of comprehensive sensing of multiple parameters (RI, magnetic field, temperature, and voltage).

## 2. Models and Theories

**Figure 1:**
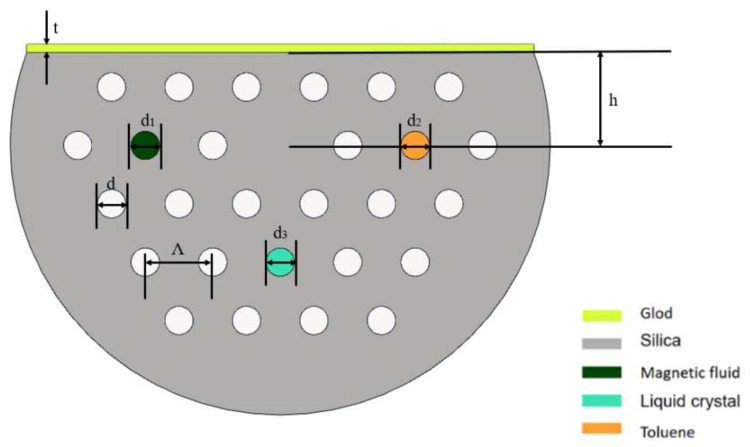
Structure diagram of the selectively filled D-shaped PCF.

The multi-parameter sensing structure based on a selectively filled D-shaped PCF is described in Figure 1. The hole spacing *Λ* = 8 µm, and air hole diameter *d* = 0.42 × *Λ*. The diameters of the air holes that are filled with MF, toluene, and LC are expressed as *d*_1_, *d*_2_ and *d*_3_, respectively, and *d*_1_ = *d*_2_ = *d*_3_ = *d*. One side of the PCF was polished, and the polishing depth *h* = 11 µm. A gold nano-film was coated on the polishing plane, and the thickness *t* = 40 nm. Unless otherwise stated, the next discussions are primarily according to these parameters. The analytical liquid is on the gold nano-film (on the D-shaped surface), so the gold nano-film can be used as an SPR layer to excite surface plasmon polarons (SPPs). When the RI of the liquid is changed, the corresponding SPR loss peak drifts, thus realizing the RI sensing measurement of the sensor. The air hole filled with MF (water-based Fe_3_O_4_), designated as channel 1 realizes the sensing measurement of the magnetic field. The air hole filled with toluene named channel 2 realizes the sensing measurement of the temperature. The air hole filled with LC, channel 3, realizes the sensing measurement of the voltage. The resonance wavelength peaks and wavelength shifts corresponding to the three channels are different, and referring to the two-parameter sensitivity matrix [32], the simultaneous sensing and detection of the magnetic field, temperature, and voltage can be achieved.

The background material of the D-shaped PCF is silicon dioxide whose RI can be obtained using the Sellmeier Equation [33]:

(1) $$n^{2}(\lambda )=1+\frac{0.6961663\lambda^{2}}{\lambda^{2}-{\left(0.0684043\right)}^{2}}+\frac{0.4079426\lambda^{2}}{\lambda^{2}-{\left(0.1162414\right)}^{2}}+\frac{0.8974794\lambda^{2}}{\lambda^{2}-{\left(9.896161\right)}^{2}}$$

where *λ* is the wavelength, whose unit is μm.

The RI of gold nano-film is calculated according to the Drude–Lorentz model [34]:

(2) $$\varepsilon_{{}_{Au}}=\varepsilon_{\infty }-\frac{\omega_{D}{}^{2}}{\omega^{2}+i\omega \gamma_{D}}-\frac{△\varepsilon \cdot \Omega^{2}{}_{L}}{(\omega^{2}-\Omega^{2}{}_{L})+i\Gamma_{L}\omega }$$

where *ω* represents the angular frequency, *ε*_∞_ represents the complex permittivity, and as *ω* → ∞, *ε*_∞_ = 5.9673. *ω*_D_ represents the plasma frequency, *γ*_D_ is the damping coefficient, Ω_L_ and Γ_L_ are the oscillator strength and spectral width, and Δε = 1.09 is the weighting coefficient. In addition, the units of *ω*_D,_ *γ*_D,_ Ω_L,_ and Γ_L_ are cm^−1^.

MF is a colloidal solution, and water-based Fe_3_O_4_ was used in this paper. Under the action of a magnetic field, the thermal and magnetic energy of nanoparticles plays a key role in forming magnetic columns in ferrofluid. The RI of MF is mainly affected by external temperature and magnetic field strength and is attributed to the formation of magnetic columns [35]. The RI can be described using the Langevin function [36]:

(3) $$n_{\mathrm{MF}}(H,T)=\left[n_{s}-n_{o}\right]\left[\coth(\alpha \frac{H-H_{c,n}}{T})-\frac{T}{\alpha (H-H_{c,n})}\right]+n_{o}$$

where *n*_s_ and *n*_o_ are the RI of magnetic field intensity in saturation and critical states, respectively, where *n*_s_ = 1.4704, *n_o_* = 1.462. *H_c,n_* is the critical magnetic field, *H* represents the applied magnetic field, and the unit is Oe, where *H* > *H_c,n_*; *T* is the thermodynamic temperature, its unit is °C, and *α* is the fitting parameter.

As a temperature-sensitive material, toluene is often used for temperature sensing, and its RI is mainly affected by temperature. The RI of toluene is calculated using the Sellmeier equation [37]:

(4) $$n\left(\lambda \right)=1.474775+\frac{6990.31}{\lambda^{2}}+\frac{2.1776\times 10^{8}}{\lambda^{4}}-\alpha \left(T-20.15\right)$$

where *λ* represents the wavelength of the incident light in μm, *T* represents the ambient temperature in °C, α = 5.273 × 10^−4^/°C, which is the temperature sensitivity coefficient.

LC is a voltage-sensitive medium, and its RI is mainly affected by voltage, followed by temperature. The RIs *n*_e_ and *n*_o_ of LC under varying temperature and wavelength can be obtained using the following formula [34]:

(5) $$n_{eo}=A_{eo}+\frac{B_{eo}}{\lambda^{2}}+\frac{C_{eo}}{\lambda^{4}}$$

where *n_e_* and *n_o_* represent the extraordinary and ordinary RI, respectively. *Ae*, *Be*, *Ce*, *Ao*, *Bo*, and *Co* are Cauchy coefficients, which are sensitive to temperature. When the temperature is 20 °C, the values of *Ae*, *Be*, *Ce*, *Ao*, *Bo*, and *Co* are 1.6993, 0.0085, 0.0027, 1.4998, 0.0067, and 0.0004, respectively. The relation of these coefficients and temperature can be obtained from [34]. When there is applied voltage, the orientation angle of the LC molecule will be deflected, and the deflection angle is expressed as follows [34]:

(6) $$\theta =\left\{\begin{array}{cc} 0 & V\leq Vc\\ \frac{\pi }{2}-2\tan^{-1}\left[\exp(-\frac{V-Vc}{30Vc})\right] & V>Vc \end{array}\right.$$

where *Vc* is the threshold voltage in *V*, and the LC molecules start to deflect when *V* > *Vc*. In addition, if the voltage *V* reaches saturation, *θ* ≈ 90°. The relationship between RI and *θ* is expressed as [34]

(7) $$n=\frac{n_{e}n_{0}}{\sqrt{n_{e}{}^{2}\cos^{2}\theta +n_{0}{}^{2}\sin^{2}\theta }}$$

The effective RI of plasma mode is greatly affected by the change of RI of liquid near the gold film but has little effect on the effective RI of the fiber core mode. Therefore, there is a resonant wavelength shift when the two modes satisfy the wave vector matching condition. Therefore, the effective RI of the liquid can be detected by measuring the offset of the resonance wavelength. The RI sensitivity is shown as follows:

(8) $$S_{n}\left(nm/RIU\right)=\frac{\Delta \lambda }{\Delta n}$$

Similarly, the RIs of the filled sensitive materials (MF, toluene, and LC) vary with external magnetic field strength, temperature, and voltage, respectively, which means that the resonance peak will shift to different wavelength positions, so the measurement of these three parameters can be realized by the resonance wavelength offsets. According to the definition, the calculation formulas for magnetic field strength, temperature, and voltage sensitivity are as follows:

(9) $$S_{H}\left(\mathrm{nm}/\mathrm{Oe}\right)=\frac{\Delta \lambda_{1H}}{\Delta H}$$

(10) $$S_{T}\left(\mathrm{nm}/T\right)=\frac{\Delta \lambda_{2T}}{\Delta T}$$

(11) $$S_{V}\left(\mathrm{nm}/V\right)=\frac{\Delta \lambda_{3V}}{\Delta V}$$

Therefore, the relationship between resonance wavelength shift, magnetic field intensity change, temperature change, and voltage change can be expressed by the sensitivity matrix:

(12) $$\left(\begin{array}{c} \Delta \lambda_{1}\\ \Delta \lambda_{2}\\ \Delta \lambda_{3} \end{array}\right)=\left(\begin{array}{ccc} \frac{\Delta \lambda_{1H}}{\Delta H} & \frac{\Delta \lambda_{1T}}{\Delta T} & \frac{\Delta \lambda_{1V}}{\Delta V}\\ \frac{\Delta \lambda_{2H}}{\Delta H} & \frac{\Delta \lambda_{2T}}{\Delta T} & \frac{\Delta \lambda_{2V}}{\Delta V}\\ \frac{\Delta \lambda_{3H}}{\Delta H} & \frac{\Delta \lambda_{3T}}{\Delta T} & \frac{\Delta \lambda_{3V}}{\Delta V} \end{array}\right)\left(\begin{array}{c} \Delta H\\ \Delta T\\ \Delta V \end{array}\right)=\left(\begin{array}{ccc} S_{1}(H) & S_{1}(T) & S_{1}(V)\\ S_{2}(H) & S_{2}(T) & S_{2}(V)\\ S_{3}(H) & S_{3}(T) & S_{3}(V) \end{array}\right)\left(\begin{array}{c} \Delta H\\ \Delta T\\ \Delta V \end{array}\right)$$

where ∆*λ*_*i*,*j*_, *i* = 1, 2, 3, *j* = *H*, *T*, *V* is the resonance wavelength offset caused by the magnetic field strength, temperature, and voltage change in the channel of the three filled materials. Δ*H*, Δ*T*, and Δ*V* are the variations of the above three parameters, respectively.

(13) $$\left(\begin{array}{c} \Delta H\\ \Delta T\\ \Delta V \end{array}\right)={\left(\begin{array}{ccc} S_{1}(H) & S_{1}(T) & S_{1}(V)\\ S_{2}(H) & S_{2}(T) & S_{2}(V)\\ S_{3}(H) & S_{3}(T) & S_{3}(V) \end{array}\right)}^{-1}\left(\begin{array}{c} \Delta \lambda_{1}\\ \Delta \lambda_{2}\\ \Delta \lambda_{3} \end{array}\right)$$

Inverting the sensitivity matrix in Equation (12), we can obtain the sensing matrix:

From Equation (13), we can obtain the changes of the magnetic field strength, temperature, and voltage by measuring the resonance wavelength offsets and thus achieve simultaneous detection.

For PCF sensors, the greater the sensitivity, the larger the full width at half maximum (FWMH) of the loss peak and the lower the resolution of the sensor. Therefore, in order to fully understand the performance of the sensor, the quality factor figure of merit (FOM) is introduced, which is as follows [38]:

(14) $$FOM=\frac{S}{FWHM}$$

Similarly, the quality factor Q is also an important parameter to characterize the sensor performance, which can be expressed as follows:

(15) $$Q=\frac{\Delta \lambda }{FWHM}$$

In the field of waveguide optics, constraint loss can be expressed as [39]:

(16) αdB/cm=8.686×2πλ×Im(neff)

where *λ* is the light wavelength, and Im(*n_eff_*) represents the imaginary part of the effective RI.

For the experiment, we prepared the proposed sensor structure using the femtosecond laser-assisted selective infiltration of PCF and the wheel polishing setup to realize the side-polishing of the PCF. Selective material filling of the PCF includes three steps: (1) conventional fusion splicing and laser cleaving, (2) selective laser drilling, and (3) infiltration. The PCF was first spliced with a section of SMF using a conventional fusion splicer. Then the spliced fiber sample was cleaved near the splicing point by the fs laser. After the laser-cleaving process, the spliced end of the PCF was fully blocked by a section of SMF. The air-holes to be selectively filled were then opened up by direct fs laser drilling from the cleaved end of the fiber sample. Finally, the drilled fiber end was immersed into the liquid to be infiltrated. Once the infiltration procedure was completed, the SMF introduced in the step 1 was cut off using a conventional fiber cleaver [40,41]. By repeating the above steps, magnetic fluid, toluene, and nematic liquid crystal were selectively filled into different cladding air holes of the PCF. After that, we used the wheel polishing setup to realize the side-polishing of the PCF. In the fiber polishing system, the grinding wheel was fastened on a 3D mechanical platform that could move along the X, Y, and Z directions. The polishing length and polishing depth were easy to set up and operate accurately via computer program. After completion of the polishing process, gold thin films were deposited on the flat surface of the D-shaped PCF by a fiber magnetron sputtering coating machine [9]. Thus, the work of fabricating the proposed sensor was accomplished.

## 3. Simulation Results and Analysis

**Figure 2:**
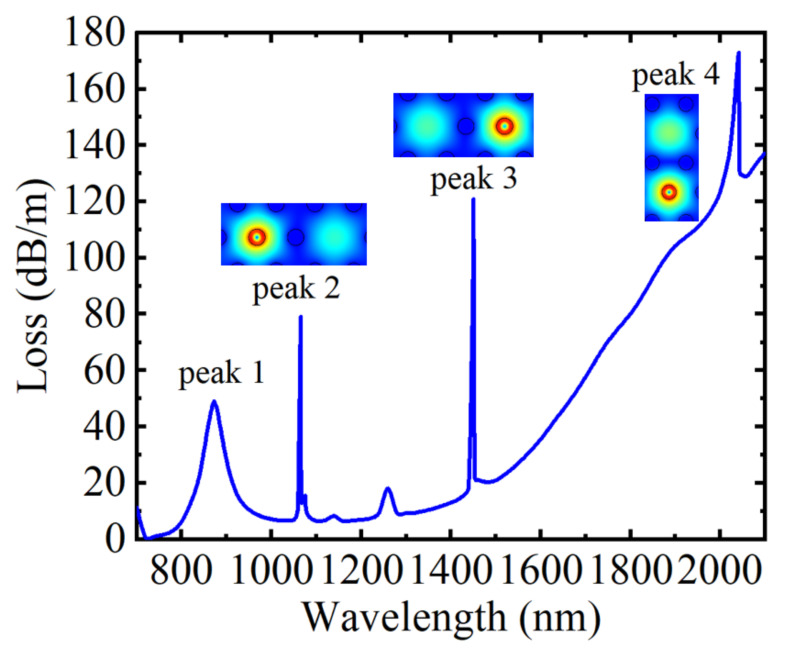
The loss spectrum of the core guide mode of the D-shaped PCF when *n* = 1.41, *H* = 250 Oe, *T* = 20 °C, and *V* = 30 V, the insets are the mode field distribution of peak 2, peak 3, and peak 4, respectively.

In this work, the sensing performances of the D-shaped PCF were numerically simulated using COMSOL Multiphysics software. Figure 2 depicts the sensing characteristic curve of the D-shaped PCF based on SPR and directional coupling when *n* = 1.41, *T* = 20 °C, *H* = 250 Oe, and *V* = 30 V. When the phase-matching condition is reached between the plasma mode and the fiber core mode, the SPR effect will occur [42]. At this time, most fiber core energies are transferred to the metal layer, and the fiber core loss increases, resulting in a resonance peak, as shown in peak 1 in Figure 2, and the resonance wavelength is λ = 873 nm. Peak 2 is a coupling peak caused by the directional coupling effect between the fundamental mode and the waveguide filled with MF. At this time, most of the energy of the fiber core is strongly coupled into the waveguide filled with MF, so there will be a loss peak, and the corresponding resonance wavelength at this time was λ = 1066 nm. Similarly, peak 3 and peak 4 are the coupling peaks caused by the directional coupling effect between the fundamental mode and the waveguide filled with toluene and LC, and the corresponding resonance wavelengths were λ = 1451 nm and λ = 2042 nm, respectively. In addition, the mode field distribution diagrams of peak 2, peak 3, and peak 4 were intercepted, respectively, as illustrated in the inset of Figure 2. It can be known from physical phenomena that the directional coupling effect will occur at the resonant wavelength. By comparing the four peaks, it can be found that the coupling strengths of the four peaks are different from each other. Therefore, the sensing of different parameters can be realized by detecting the wavelength shift corresponding to different loss peaks.

**Figure 3:**
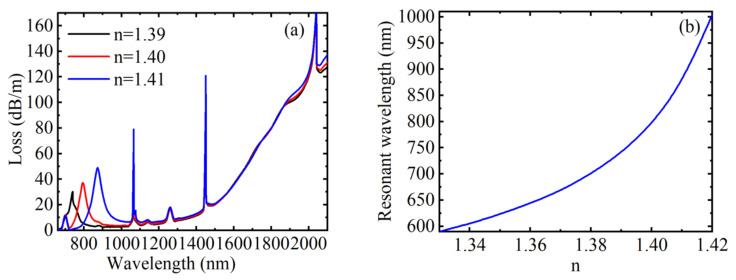
When *H* = 250 Oe, *T* = 20 °C, and *V* = 30 V, under different RIs, (**a**) the loss spectrum of the core guide mode, (**b**) the variation relationship between the resonance wavelength corresponding to peak 1 and RI.

Figure 3a shows the loss spectrum at which the RI of the analytical solution is 1.39, 1.40, and 1.41 when *H* = 250 Oe, *T* = 20 °C, and *V* = 30 V, respectively. From the simulation results in Figure 3a, it can be found that the resonant wavelength redshifts and the coupling strength increases when the RI of the analyzed liquid increases. This is because the change of RI of liquid near the gold film will affect the effective RI of the excited surface plasma wave. When *n* changed initially from 1.39 to 1.40, the displacement of the resonant wavelength was calculated as 54 nm, and when it changed to 1.41, the displacement of resonant wavelength increased to 79 nm. In addition, changing the RI of the analytical solution does not affect the position of other arbitrary peaks, this is because the RI change of the liquid near the gold film has no effect on the RIs of the MF, toluene, and LC. Moreover, the other peaks have larger absorption intensity, and the peaks are narrower. When the magnetic field, temperature, and voltage remain unchanged, the relationship between the resonant wavelength corresponding to the SPR-based loss peak and *n* is demonstrated in Figure 3b. From Figure 3b, we can observe that the resonant wavelength changed linearly with the RI first and then increased sharply when the RI was in the range of 1.33~1.42. The average RI sensitivity in this range was 4600 nm/RIU. The resonance wavelength changed from 590 to 1004 nm, the FWHM changed from 25 to 70 nm, the calculated RI sensitivity varied from 1500 to 13,100 nm/RIU, the FOM changed from 60 to 187.14 RIU^−1^, and Q changed from 0.6 to 1.87 when the RI changed from 1.33 to 1.42. Therefore, the RI sensitivity, FOM and Q value all increase with the increase of RI, and the RI sensing of the device is not influenced by the external magnetic field, temperature, and voltage.

**Figure 4:**
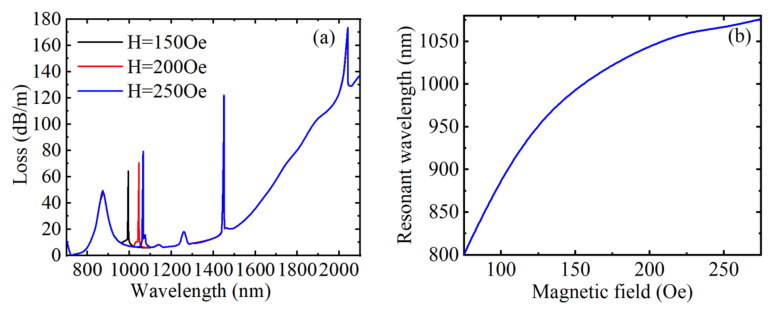
When *n* = 1.41, *T* = 20 °C, and *V* = 30 V, under different magnetic field strengths, (**a**) the loss spectrum of the core guide mode, (**b**) the variation relationship between the resonance wavelength corresponding to peak 2 and the magnetic field strength.

Figure 4a shows the loss spectrum at the magnetic field strengths of 150, 200, and 250 Oe when *n* = 1.41, *T* = 20 °C, and *V* = 30 V, respectively. When the magnetic field strength increased, the directional coupling peak caused by the MF filled with air hole redshifted, and the peak value also increased gradually. The reason is that changes in the magnetic field alter the RI of the channel, thereby affecting the RI of the directionally coupled waveguide mode, resulting in a change in energy coupling. At the same time, it can be clearly found that the shift of the resonance wavelength is reduced, which can be obtained from Figure 4b. Similarly, changing the magnetic field strength did not affect the position of any other peaks; this is because the change of magnetic field intensity has no effect on the RIs of the liquid near the gold film, toluene and LC. Figure 4b describes the relationship between the resonance wavelength and the magnetic field strength. When RI, temperature, and voltage remain unchanged, the resonance wavelength first changes linearly with the magnetic field strength and then gradually tends to be saturated. According to Figure 4b, when the magnetic field strength is in a smaller range, the linearity is better, the shift of the resonance wavelength with the magnetic field is more obvious, and the magnetic field sensitivity is higher. The average magnetic field sensitivity was 1.375 nm/Oe in the range of 75~275 Oe. The resonance wavelength changed from 801 to 1076 nm, the FWHM changed from 12 to 4 nm, the calculated magnetic field sensitivity varied from 3.6 to 0.4 nm/Oe, the FOM changed from 0.3 to 0.1 Oe^−1^, and Q changed from 7.5 to 2.5 when the magnetic field strength changed from 75 to 275 Oe. The magnetic field sensitivity, FOM, and Q value all decreased with the increase of magnetic field strength. In conclusion, when the structural parameters were unchanged, the magnetic field sensing of the device was not affected by the external RI, temperature, and voltage.

**Figure 5:**
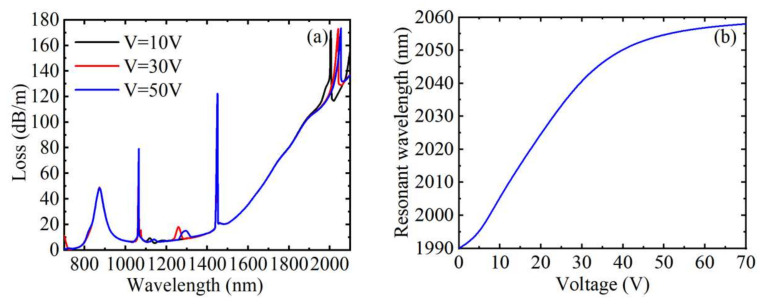
When *n* = 1.41, *T* = 20 °C, and *H* = 250 Oe, under different voltages, (**a**) the loss spectrum of the core guide mode, (**b**) the variation relationship between the resonance wavelength corresponding to peak 4 and the voltages.

Figure 5a shows the loss spectrum with the voltages of 10, 30, and 50 V when *n* = 1.41, *H* = 250 Oe, and *T* = 20 °C, respectively. As shown in Figure 5a, the directional coupling peak caused by the LC filled with air hole redshifted with the increase of voltage, and the drift of the resonance wavelength decreased, but the coupling strength changed little. The analysis of changing the magnetic field strength showed that the RI of the channel will change with the change of the voltage, which will affect the effective RI of the directional coupled waveguide mode, resulting in a change in the coupling of energy. In addition, changing the magnitude of the voltage does not affect the position of any other peaks, this is because the change of voltage has no effect on the RIs of liquid near the gold film, toluene and MF. Figure 5b plots the relationship between the resonance wavelength and the external voltage. Clearly, in the voltage range of 0~50 V, the resonant wavelength becomes more sensitive and has a good linearity with the increase of the voltage. When the resonance wavelength no longer changes, the voltage reaches saturation state. The average voltage sensitivity was 0.971 nm/V in the range of 0~70 V. The resonance wavelength changed from 1990 to 2058 nm, the FWHM changed from 9 to 16 nm, the calculated voltage sensitivity varied from 1.6 to 0.1 nm/V, the FOM changed from 0.178 to 0.006 V^−1^, and Q changed from 1.78 to 0.06 when the voltage changed from 0 to 70 V. The voltage sensitivity, FOM, and Q value all decreased with the increase of voltage. In conclusion, the voltage sensing of the device was not influenced by the external RI, magnetic field, and temperature.

**Figure 6:**
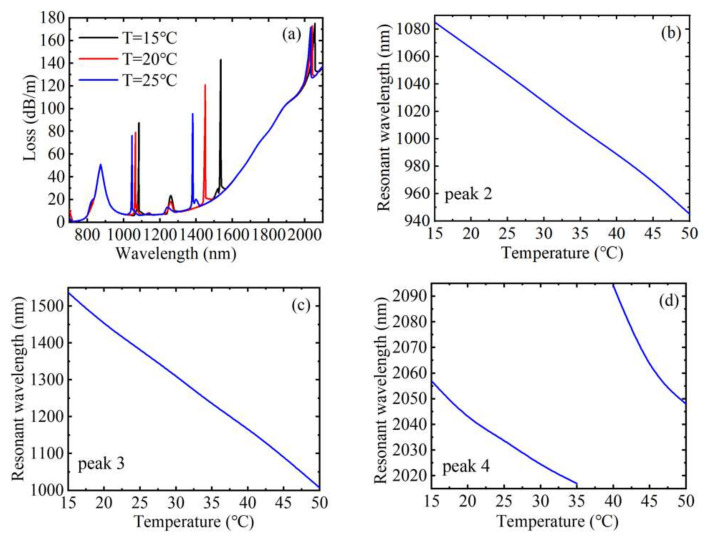
(**a**) The loss spectrum of the core guide mode with three different temperatures when *n* = 1.41, *H* = 250 Oe, and *V* = 30 V. The variation relationship between the resonant wavelength corresponding to different peaks and temperature: (**b**) peak 2, (**c**) peak 3, and (**d**) peak 4.

Figure 6a shows the loss spectrum at the temperatures of 15, 20, and 25 °C when *n* = 1.41, *H* = 250 Oe, and *V* = 30 V, respectively. From Figure 6a, we can see that when the temperature increases gradually, except for the loss peak caused by SPR, which does not shift, the other three loss peaks are blue-shifted, and the coupling strength decreases. This is because the RI of the liquid near the gold film is not affected by temperature, while the RIs of MF, toluene, and LC all change with temperature, resulting in a shift in the coupling peak. In addition, the resonance wavelength corresponding to peak 3 has the largest shift, that is, the temperature sensitivity is the largest. Figure 6b–d plot the variation curve of the resonance wavelengths corresponding to peak 2, peak 3, and peak 4 with the temperature, respectively. It can be inferred from the figure that the resonant wavelengths corresponding to the coupling effect caused by the air holes filled with MF and toluene are all linearly related to temperature, while the resonant wavelength corresponding to the coupling effect caused by the air hole filled with LC is linear in the range of 15~35 and 40~50 °C. According to the calculation, the temperature sensitivities corresponding to peak 2 and peak 3 were 4 and 15.143 nm/°C, respectively, and the sensitivity corresponding to peak 4 was 2 and 4.6 nm/°C in the two temperature ranges, respectively. Since toluene is more sensitive to temperature, the air hole filled with toluene was used as the temperature sensing channel. The resonance wavelength changed from 1538 to 1008 nm, the FWHM changed from 7 to 3 nm, the calculated temperature sensitivity varied from 17.2 to 16.8 nm/°C, the FOM changed from 2.46 to 5.6 °C^−1^, and Q changed from 12.29 to 28 when the temperature changed from 15 to 50 °C. The FOM and Q value all increased with the increase of temperature. According to Equations (12) and (13), the sensing matrix for detecting changes in external magnetic field strength, temperature and voltage can be obtained. When the temperature was 15~35 and 40~50 °C, respectively,

(17) $$\begin{array}{cc} \left(\begin{array}{c} \Delta H\\ \Delta T\\ \Delta V \end{array}\right) & ={\left(\begin{array}{ccc} 1.375 & 4 & 0\\ 0 & 15.143 & 0\\ 0 & 2 & 0.971 \end{array}\right)}^{-1}\left(\begin{array}{c} \Delta \lambda_{1}\\ \Delta \lambda_{2}\\ \Delta \lambda_{3} \end{array}\right)\\ & =\left(\begin{array}{ccc} 0.727 & -0.192 & 0\\ 0 & 0.066 & 0\\ 0 & -0.136 & 1.030 \end{array}\right)\left(\begin{array}{c} \Delta \lambda_{1}\\ \Delta \lambda_{2}\\ \Delta \lambda_{3} \end{array}\right) \end{array}$$

(18) $$\begin{array}{cc} \left(\begin{array}{c} \Delta H\\ \Delta T\\ \Delta V \end{array}\right) & ={\left(\begin{array}{ccc} 1.375 & 4 & 0\\ 0 & 15.143 & 0\\ 0 & 4.6 & 0.971 \end{array}\right)}^{-1}\left(\begin{array}{c} \Delta \lambda_{1}\\ \Delta \lambda_{2}\\ \Delta \lambda_{3} \end{array}\right)\\ & =\left(\begin{array}{ccc} 0.727 & -0.192 & 0\\ 0 & 0.066 & 0\\ 0 & -0.313 & 1.030 \end{array}\right)\left(\begin{array}{c} \Delta \lambda_{1}\\ \Delta \lambda_{2}\\ \Delta \lambda_{3} \end{array}\right) \end{array}$$

In conclusion, the sensor based on the selectively filled D-shaped PCF proposed in this paper can not only detect the four parameters of RI, magnetic field strength, temperature, and voltage simultaneously but also has high sensitivity.

Next, we discuss the structural parameters’ influence on the sensing characteristics of the selective filled D-shaped PCF in this paper. The sensing performances can be changed by optimizing the structural parameters to optimize performance. The RI of the analysis solution was fixed at 1.41, the magnetic field strength was 250 Oe, the temperature was 20 °C, and the voltage was 30 V.

**Figure 7:**
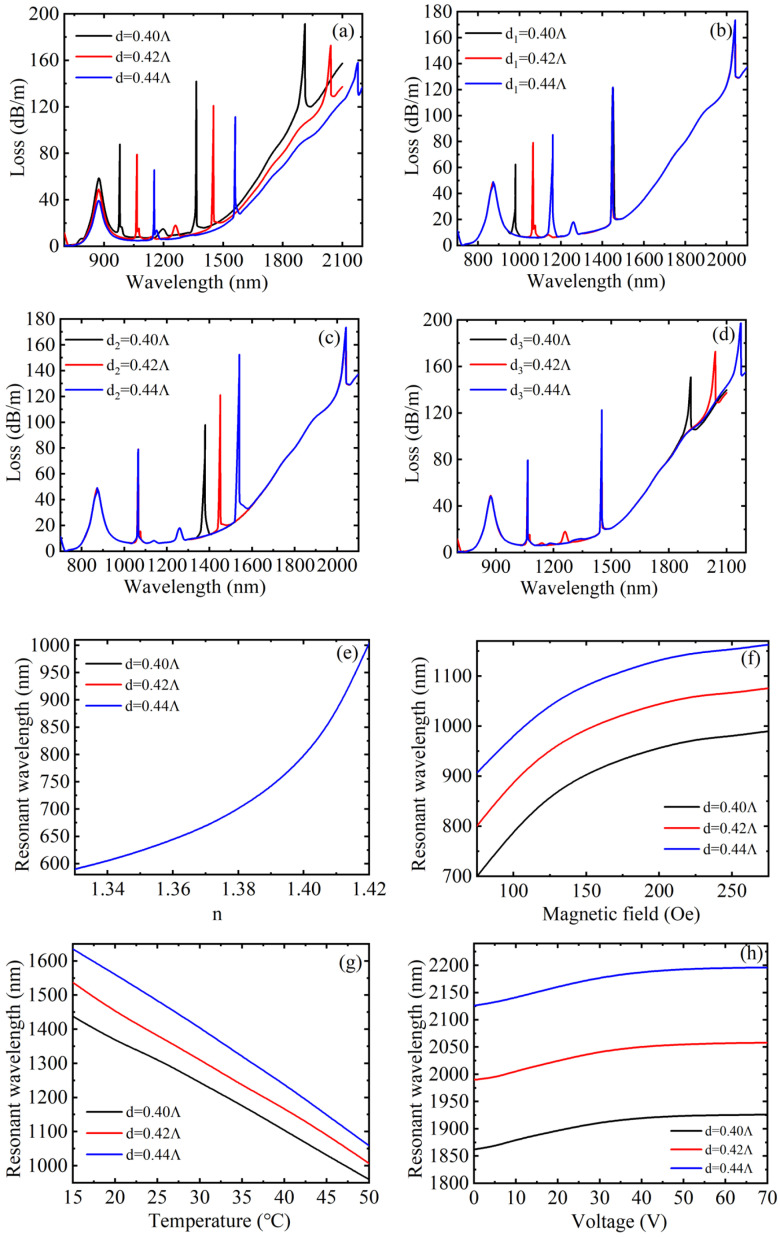
The loss spectrum of the core guide mode under different air hole diameters (**a**) *d,* (**b**) *d*_1_*,* (**c**) *d*_2_*,* and (**d**) *d*_3_. The variation relationship between the resonance wavelength and the external parameters under different air hole diameters *d* (**e**) RI, (**f**) magnetic field, (**g**) temperature, and (**h**) voltage.

When other structural parameters of the D-shaped PCF remained unchanged, core loss spectra under different air hole diameters *d*, *d*_1_, *d*_2_ and *d*_3_ are described in Figure 7a–d, respectively. As observed, when the air hole diameter *d* increased, the position of the resonance peak caused by SPR did not change and the FWHM decreased, while the other three coupling peaks due to directional coupling were all redshifted, and the FWHM of peak 2 and peak 3 both remained unchanged basically, but the FWHM of peak 4 increased, and the peak value of the four peaks decreased accordingly. This is because the equivalent RI of the cladding will decrease as the air hole diameter increases so that the RI difference between the fiber core and the cladding will increase, the fiber core area will have a stronger ability to limit the energy, resulting in a reduction in the loss of the fiber core guide mode. Furthermore, when only the air hole diameter *d*_1_ changed, it was found that only the position and value of the corresponding coupling peak changed and the other three peaks did not change. When *d*_1_ increased, the corresponding coupling peak redshifted, and the peak value increased simultaneously. The reason is that the real part of the effective RI of the waveguide will increase when air hole diameter increases, which will cause the redshift of the wave vector matching point. Thus, the air hole diameter can be adjusted as required to change the wavelength position when directional coupling occurs. The analysis of changing the air hole diameter *d*_2_ and *d*_3_ was the same as that of *d*_1_. To obtain the air hole diameter’s influence on the sensing characteristics in a more specific manner, Figure 7e–h were plotted. Figure 7e–h describe the variation of the resonance wavelength with RI, magnetic field, temperature, and voltage under three different air hole diameters *d*, respectively. As can be obtained from Figure 7e, the three variation curves basically coincided with each other under different air hole diameters, indicating that the air hole diameter has little influence on RI sensitivity. Considering that the position of the corresponding loss peak remains unchanged and the FWHM decreases, the FOM and Q values both increase. In Figure 7f, the three curves are basically parallel, indicating that the air hole diameter also has little influence on the magnetic field sensitivity. Similarly, considering that the variation of the corresponding loss peak drift is the same and there is no change in the FWHM, the FOM and Q value both remain the same. It can be seen from Figure 7g that when the air hole diameters *d* was 0.40 *Λ*, 0.42 *Λ,* and 0.44 *Λ* (*Λ* = 8 µm), the corresponding resonant wavelengths were basically linear with temperature, and the sensitivity was calculated as 13.657, 15.143, and 16.457 nm/°C, respectively. It was found that the temperature sensing sensitivity of the sensor became higher when *d* increased. Therefore, the temperature sensitivity can be improved by increasing the size of *d*. Moreover, the shift of the corresponding resonant peak increases, while FWHM remains the same, so FOM and Q values both increase. In Figure 7h, the analysis is the same as that of Figure 7f. The difference is that the FWHM increases, resulting in a decrease in FOM and Q value. The effect of air hole diameter variation on sensitivity is shown in Table 1.

**Figure 8:**
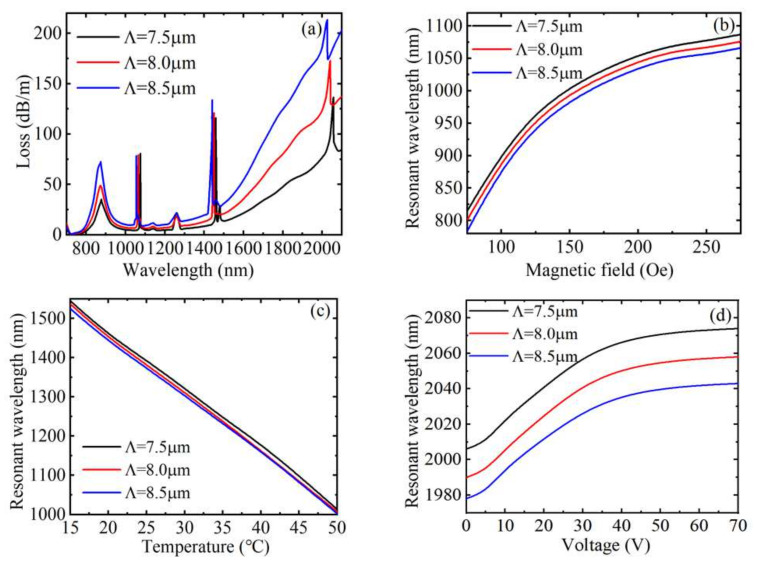
(**a**) The loss spectrum of the core guide mode with different hole spacing *Λ*. The variation relationship between the resonance wavelength and the external parameters (**b**) magnetic field, (**c**) temperature, and (**d**) voltage.

Figure 8a indicates the loss spectrum when the hole spacing *Λ* was 7.5, 8.0, and 8.5 µm, respectively. As described in Figure 8a, as *Λ* increased, the coupling intensity of the loss peak caused by SPR increased and the FWHM also increased, but the position of the resonance wavelength was hardly affected, indicating that the hole spacing has little effect on the RI sensitivity, and the FOM and Q values both decrease. The other three loss peaks all blue-shift with the increase of the hole spacing *Λ*, which has little impact on the coupling strength of the loss peaks caused by filling with MF, but the coupling strengths of the loss peaks caused by filling toluene and LC all increase, and the variations of the three loss peaks drift are not much different. Moreover, the FWHM of the loss peaks caused by filling with MF and toluene remains the same, but FWHM of the loss peak caused by filling with LC increases. The effects of hole spacing on magnetic field sensitivity, temperature sensitivity, and voltage sensitivity are shown in Figure 8b–d. In Figure 8b, the three variation curves are basically parallel. In Figure 8c, the three curves are basically coincident. Figure 8d is the same as Figure 8b. In conclusion, the hole spacing has little effect on the RI, magnetic field, temperature, and voltage sensitivity. The FOM and Q value of the loss peaks caused by filling with MF and toluene shows no change, while the FOM and Q value of the loss peaks caused by filling with LC decreases. Therefore, the sensitivity, FOM, and Q value cannot be improved by optimizing the hole spacing. The effect of hole spacing variation on sensitivity is shown in Table 2.

**Figure 9:**
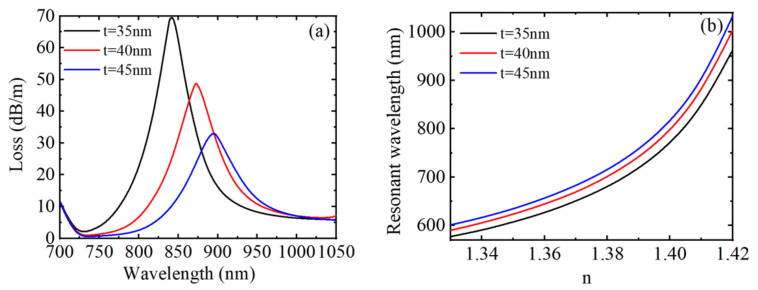
(**a**) The loss spectrum of the core guide mode under different metal nano-film thicknesses. (**b**) The variation relationship between the resonance wavelength and *n*.

Figure 9a describes the loss spectrum under three different metal nano-film thicknesses. As shown in Figure 9a, the increase of the metal nano-film thickness leads to the redshift of the peak wavelength, the decreases of the resonance peak value and the increase of FWHM. Furthermore, the resonance wavelength shifted from 842 to 895 nm when metal nano-film thickness increased from 35 to 45 nm. Figure 9b describes the relationship curve of the resonance wavelength with RI. The three variation curves are basically parallel, the variations of the loss peak drift are not much different, and the RI sensing sensitivity can be calculated by the slope of the curve. As seen in Figure 9b, the sensitivity of the D-shaped PCF with respect to the RI sensing has a small difference under different gold nano-film thicknesses, while the FOM and Q values decrease, so the change of metal film thickness has little influence on the sensitivity of RI sensing except for the sharpness, peak position, and height of the SPR peak. Therefore, changing the metal nano-film thickness cannot enhance the RI sensitivity but can adjust the FOM and Q value.

**Figure 10:**
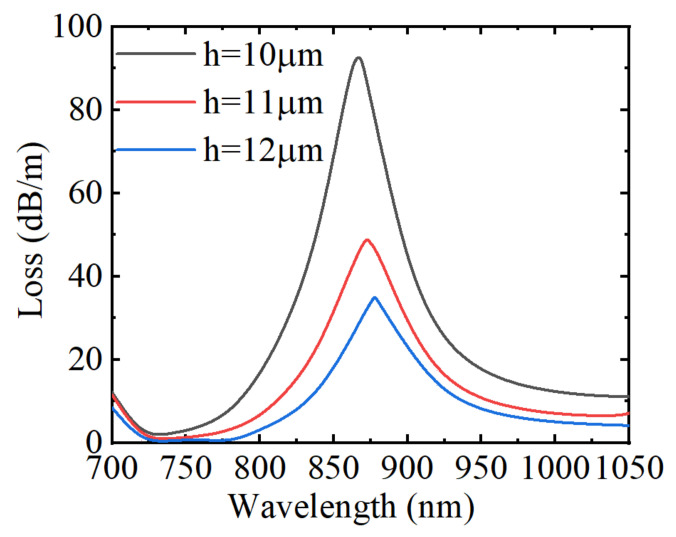
The loss spectrum of the core guide mode under three polishing depths.

Figure 10 depicts the loss spectrum when the polishing depth *h* was 10, 11, and 12 µm, respectively. It can be found that when other parameters remain unchanged, the change of the polishing depth *h* has little impact on the position of the resonance wavelength, but with the gradual increase of the *h* value, the absorption intensity of the loss peak decreases and FWHM increases. This is because increasing the polishing depth will reduce the evanescent wave energy of the fiber core reaching the surface of the metal layer, then the SPR effect will become weaker, but it will not change the dispersion curve of the plasmon mode on the surface of the metal layer. Therefore, it has little influence on the effective RI of the fiber core. Accordingly, the position of the resonance wavelength will not change basically. Therefore, changing the polishing depths cannot enhance the RI sensitivity but can adjust the FOM and Q value.

In order to prove that the designed sensor has good sensing performance, the performance of the RI, magnetic field, temperature, or voltage sensor of the structure proposed in this paper is compared with other structures in Table 3. Compared with other types of sensors, the sensor structure proposed in this paper not only achieves four-parameter sensing but also has higher sensing sensitivity, which plays an important role in the field of environmental detection and integrated optoelectronic devices.

## 4. Conclusions

In summary, we proposed and numerically investigated a multi-parameter sensor based on a selectively filled D-shaped PCF according to the sensing mechanisms of SPR and directional coupling effect, which can simultaneously detect four parameters of RI, magnetic field, temperature, and voltage. The advantage of this sensor is that it can effectively solve the problem of cross-sensitivity among the four parameters and the limitation of single-parameter detection. Therefore, the sensor provides a new idea for the multi-functional sensing field. According to the simulation analysis, when the refractive index range was 1.33~1.42, the average refractive index sensitivity of the sensor was 4600 nm/RIU, and when the magnetic field range is 75~275 Oe, the magnetic field sensitivity can be up to 1.375 nm/Oe. Moreover, when the temperature range was 15~50 °C, the temperature sensitivity was 15.143 nm/°C, and when the voltage range was 0~70 V, the voltage sensitivity was 0.971 nm/V, higher than some other published sensors [32,43,44,45,46]. Therefore, it can be concluded that the sensor based on the material-selectively filled D-shaped PCF designed in this paper has important application value in fiber communication, fiber sensing, and many other fields.

## Figures and Tables

**Table 1:** Effect of air hole diameter variation on sensitivity.

Air Hole Diameter (µm)	Resonance Wavelength (nm)				Sensitivity			
	Peak 1	Peak 2	Peak 3	Peak 4	RI (nm/RIU)	Magnetic Field (nm/Oe)	Temperature (nm/°C)	Voltage (nm/V)
0.40*Λ*	874	980	1366	1912	4600	1.450	13.657	0.928
0.42*Λ*	873	1066	1451	2042	4600	1.375	15.143	0.971
0.44*Λ*	873	1153	1561	2178	4600	1.280	16.457	1.014

**Table 2:** Effect of hole spacing variation on sensitivity.

Hole Spacing (µm)	Resonance Wavelength (nm)				Sensitivity			
	Peak 1	Peak 2	Peak 3	Peak 4	RI (nm/RIU)	Magnetic Field (nm/Oe)	Temperature (nm/°C)	Voltage (nm/V)
7.5	877	1077	1459	2058	4600	1.360	15.200	0.985
8.0	873	1066	1451	2042	4600	1.375	15.143	0.971
8.5	875	1056	1441	2027	4600	1.410	14.943	0.942

**Table 3:** Performance comparison of different sensors.

Sensor Type	RI Sensitivity (nm/RIU)	Magnetic Field Sensitivity (nm/Oe)	Temperature Sensitivity (nm/°C)	Voltage Sensitivity (nm/V)
Graphene-gold coated structure [43]	4200	-	-	-
Based on Mach–Zehnder interferometer [44]	-	0.072	−0.080	-
Double-core D-shaped structure [45]	-	0.0779	1.151	-
D-shaped structure [32]	-	0.14274	−0.229	-
Based on reflective Fabry–Perot [46]	-	1.02602	-	-
Structure of this work	4600	1.375	15.143	0.971

## Data Availability

Data sharing is not applicable to this article.
